# Comparison of Human and Porcine Natural Tooth Fluorescence—A Scoping Study to Inform Research on Dental Materials and Forensic Dentistry

**DOI:** 10.1002/cre2.70052

**Published:** 2024-12-12

**Authors:** Thomas Corfield, Denice Higgins

**Affiliations:** ^1^ Adelaide Dental School The University of Adelaide Adelaide South Australia Australia; ^2^ South Australian Forensic Odontology Unit, Adelaide Dental School The University of Adelaide Adelaide South Australia Australia

## Abstract

**Objectives:**

Understanding human tooth structure fluorescence aids clinical and forensic dentistry, enabling tissue/material differentiation and the creation of esthetic restorative materials. Material manufacturers seek to replicate natural tooth fluorescence, necessitating the development of novel techniques to detect them. Procuring human teeth for research is challenging due to ethical and infection control standards, prompting a search for alternative models.

**Material and Methods:**

This study compares visible light‐induced fluorescence of porcine and human teeth to assess the value of porcine teeth as human analogs. Using a pulsed laser, an optimal fluorescence‐inducing wavelength was determined, followed by comparing fluorescence spectra between species.

**Results:**

Luminescence sensitivity and lifetimes were comparable between species, but spectral geometry differed.

**Conclusion:**

Porcine teeth, commonly used for dental material investigations, may not be suitable for dental fluorescence studies due to spectral differences. Accurately mimicking human tooth fluorescence remains complex. Further research is needed to develop reliable alternatives for dental fluorescence investigations that will advance clinical and forensic dentistry.

## 1 Introduction

Ultraviolet light has been used for many years to differentiate tooth‐colored dental materials from natural teeth in both clinical and forensic dentistry. However, the use of ultraviolet light requires minimal ambient lighting and is potentially hazardous to users. Moreover, as dental material manufacturers strive to mimic the natural tooth's ultraviolet fluorescence, this method of differentiation may be less effective as they continue to evolve (Volpato, Pereira, and Silva 2018). Dental fluorescence is a result of the absorption of invisible ultraviolet light with visible blueish‐white light re‐emitted (Spitzer and Bosch Ten 1976). Ultraviolet fluorescence of natural tooth gives it a luminescence that adds an overall vitality and brightness by adding fluorescing blue light to existing reflections. Materials manufacturers have focused on ultraviolet excitatory wavelengths to mimic this natural fluorescence, as these are the main sources of tooth luminescence in daylight. As non‐ultraviolet wavelengths are considered less significant contributors to tooth fluorescence, they are likely to be ignored by manufacturers and may be exploited as a new means of restoration detection. Moreover, the examination of teeth with wavelengths longer than ultraviolet will be less hazardous to operators.

Fluorescence is a photoelectric effect that occurs when valence electrons of a material absorb certain wavelengths of incident light and spontaneously re‐emit them at longer wavelengths. These longer wavelengths correlate to lower energy, as some incident light's energy is consumed to facilitate the process. Fluorescence is the immediate re‐emission of light, and phosphorescence is delayed re‐emission, with both being examples of luminescence (Hoerman and Mancewicz 1964). Tooth fluorescence will vary depending on the excitatory wavelength they are exposed to because their fluorophores, the specific substances that undergo the effect, have differing electrical configurations. These include the excitatory wavelength inducing the effect, the resultant re‐emitted wavelengths and their intensity, changes in intensity over prolonged excitation (known as luminosity sensitivity), and how long the fluorescence takes to decay (known as fluorescence lifetime) (Turoverov et al. 1998; Nishijima 1970; Lakowicz 2006).

Many dental diagnostic tools make use of visible light excitatory wavelengths. However, these tools tend to detect biological markers associated with decay activity rather than tooth mineral structure. Diagnodent, for example, uses 655 nm excitatory light to induce 900 nm near‐infrared fluorescence in the metabolic by‐products of cariogenic bacteria as a qualitative measure of decay (Lussi, Hibst, and Paulus 2004). In quantitative induced light fluorescence (QLF), blue excitatory light is used to induce green fluorescence in sound dental tissues and red fluorescence in metabolic cariogenic by‐products. QLF also assesses the extent of the diseased tooth by measuring the reduction in green caused by light scattering through the damaged mineral structure. Fluorescence Aided Caries Excavation (FACE) exploits similar wavelengths to QLF, although without assessing this scattering (Alammari et al. 2013; Lennon et al. 2009).

These diagnostic tools were developed from visible light dental fluorescence investigations following the commercial availability of various lasers. Taubinsky et al. demonstrated that human tooth structure fluoresces 700 nm (red) under 633 nm wavelength illumination and that the fluorescence of enamel, dentine, and cementum, including cariously affected examples, are identical, although differ markedly in intensity (Taubinsky et al. 2000). Sundstrom et al. used three laser wavelengths to examine the fluorescence variability between intact and early, non‐cavitated carious lesions. They found that 488 nm (cyan) incident light‐induced 540 nm (green) fluorescence in dentine and enamel, with caries eliciting stronger fluorescence, although with a slight red shift, meaning the spectroscopic shape remains the same but is shifted slightly toward the red. They also found that 515 nm (green) laser light also induced 540 nm (green) fluorescence, although with less difference between intact and carious enamel. They concluded that 488 nm was best at inducing fluorescence disparity between initial enamel caries and intact enamel (Sundström et al. 1985).

Although debate continues regarding the identity of dental fluorophores, it is accepted that dental fluorescence arises from a number of components and complexes (Matsumoto, Kitamura, and Araki 2001). It is also accepted that dental investigations into fluorescence are complicated by tooth age and even temperature and by the tissue components being examined (Booij and Bosch 1982). Enamel, for example, tends to have more fluorescence consistency among individuals, although this is affected by environmental surface mineral accumulations because of its high apatite component (Björkman, Sundström, and ten Bosch 1991; Kvaal and Solheim 1989, 1995). Dentine, however, may be more variable because of a greater diversity of genetically dependent organic components. It has also been shown that tooth fluorescence decreases as tooth mineralization increases, which may account for the decreased fluorescence observed in older teeth (Björkman, Sundström, and ten Bosch 1991; Kvaal and Solheim 1989, 1995). Fluorescence intensity even varies with the tooth surface. The cervical areas of teeth, for example, fluoresce with greater intensity than cuspal areas, presumably because there is less enamel cervically to obfuscate the excitatory light upon underlying dentine and its resultant fluorescence (Spitzer and Bosch Ten 1976; Hall, Hefferren, and Olsen 1970; Horibe et al. 1974). Even tooth whitening has been shown to alter tooth fluorescence intensity (Caneppele, Torres, and Bresciani 2015). Further complications arise when recognizing that while some studies have demonstrated no differences in fluorescence between tooth types from the same individual, other studies have found differences in both intensity and color (Dickson et al. 1952; Matsumoto, Kitamura, and Araki 1999). However, minimal difference has been shown between upper and lower teeth from the same individual or between sexes (Kvaal and Solheim 1989, 1995; Matsumoto, Kitamura, and Araki 1999).

These fluorescence inconsistencies aside, mammalian teeth are commonly used in dental research. Bovine teeth, for example, have been used as human analogs for many years (Yassen, Platt, and Hara 2011), and porcine teeth are often used as substitutes for human teeth in in vitro experiments (Olek, Klimek, and Bołtacz‐Rzepkowska 2020; Fonseca et al. 2004). Indeed, these studies conclude that teeth of breeding animals, in particular, such as cows, sheep, and pigs, are ideal because not only are they numerous and available, but they allow teeth to be of consistent age and dietary background, both of which can influence the extent of enamel mineralization and chemical composition (Dutra‐Correa, Anauate‐Netto, and Arana‐Chavez 2007; Pashley et al. 1988; Tanaka et al. 2008). Moreover, the use of animal teeth eliminates issues associated with cross‐infection, particularly in the post‐Covid world, a consideration currently highlighted by Ethics Committees and one that has led to the International Standards Organization encouraging researchers to use such teeth rather than extracted human teeth (Tanaka et al. 2008).

It is important to note, however, that most studies using porcine or bovine teeth as human analogs do not explore dental fluorescence. Rather, they assess the properties of dental restorative materials; that is, they utilize the physical similarities of these species' dental microanatomy and chemistry to investigate, for example, dental adhesive systems (Soares et al. 2016). While such investigations have demonstrated that the physical characteristics of human, bovine, and porcine teeth are similar, this does not necessarily translate to similar fluorescence characteristics. Although physical differences primarily involve crystal prism shape and dimensions, with porcine teeth having a similar mineral structure to human teeth, chemically, there is greater variability between species (Olek, Klimek, and Bołtacz‐Rzepkowska 2020; Lopes et al. 2006). For example, thermally denatured human enamel has a greater chemical similarity to synthetic hydroxyapatite than to bovine or porcine enamel, while chemically denatured human enamel demonstrates the opposite (Björkman, Sundström, and ten Bosch 1991; Teruel et al. 2015).

Clearly, caution is required when extrapolating these comparisons to investigations of dental fluorescence, not least because fluorophores behave differently when in solution than as solids. For example, solutions of fluorophores may form condensing aggregates with different molecular arrangements that directly influence their electrical configurations and resultant fluorescence characteristics (Olympus Life Science 2024). In addition, fluorescence detection itself can be influenced by the observation technique used and the geometry of the sample relative to the emission detector (Vilchez et al. 1993; Samokhvalov 2020). Solutions tend to be observed via light transmission techniques, for example, whereas solids are often observed via reflection, resulting in fundamentally inconsistent comparisons of spectra. It has, therefore, been recommended that differences in the organic and mineral components of species' teeth, along with the sampling and observation methods, be taken into consideration when interpreting any fluorescent results obtained from mammalian dental analogs (Ortiz‐Ruiz et al. 2018).

Nevertheless, with regard to dental fluorescence, and while it is generally accepted that there is little difference between the ultra‐violet dental fluorescence of different species, particularly human and bovine teeth (Spitzer and Bosch Ten 1976), this paper aims to determine whether the same is true of visible light fluorescence in porcine teeth. For the reasons outlined above, such validation might assist future research into dental fluorescence by validating the use of porcine teeth as suitable human substitutes.

## 2 Aims

This study aims to determine whether a porcine tooth can be an acceptable analog for a human tooth in studies of dental fluorescence by comparing four fluorescence characteristics:

1. *Excitation wavelength*. Determine whether the teeth share a common excitatory wavelength that induces maximum fluorescence.
2. *Fluorescence lifetime*. Compare the fluorescence emission lifetimes of both teeth following cessation of excitation.
3. *Fluorescence spectra*. Compare the fluorescence spectral profile that arises from excitation at this wavelength.
4. *Luminosity sensitivity*. Determine whether prolonged exposure at this wavelength increases or decreases the fluorescence.

## 3 Materials and Methods

### 3.1 Informed Consent/Ethics Approval

The University of Adelaide classifies research that carries only negligible risk and involves the use of existing data that contains only non‐identifiable data about human beings as exempt from ethical review. The study was granted an exemption from requiring ethics approval by the University of Adelaide Human Research Ethics Committee, as it did not involve human participants or animals.

### 3.2 Samples

Six archival human teeth from unknown individuals were obtained, along with six porcine teeth retrieved from abattoir pigs. For both species, these consisted of two first molars, two premolars and two incisors. While the human teeth were from individuals of unknown age, demography, and ethnicity, the six porcine teeth were from three 6‐month‐old pigs from one abattoir.

The porcine teeth were dry autoclaved, while the human samples, being archival, were not. Dry autoclaving was chosen because its high heat and pressure would denature proteins in the pulp tissue, potentially making the organic content more similar to that of aged specimens (Salem‐Milani et al. 2015). Following this, all samples were soaked in distilled water for 24 h before being scrubbed with a toothbrush to remove any residual organic deposits on the porcine teeth and to rehydrate any external adherences for removal on the archival human teeth.

**Figure 1:**
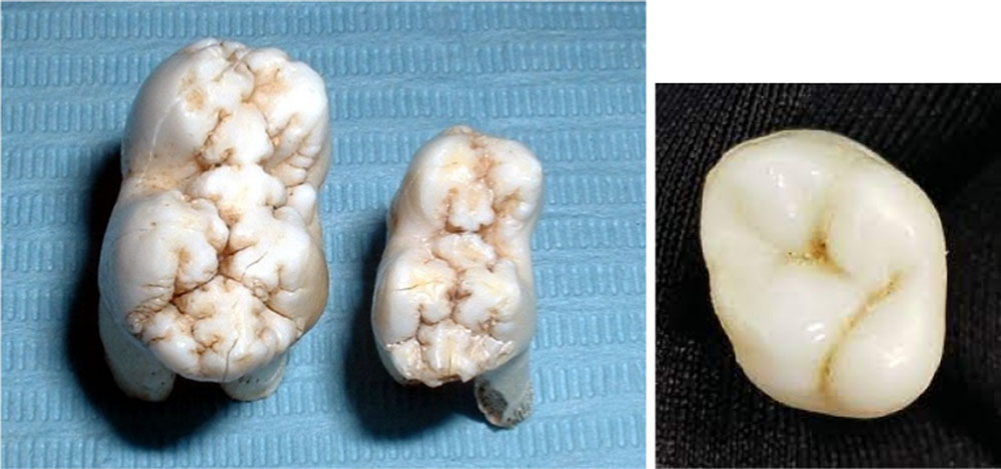
Porcine molar and premolar (left) and human third molar (right).

Porcine molars and premolars are different‐sized versions of each other, both have greater (see Figure 1). Porcine and human incisors have similar labial surfaces, while Human molars and porcine molars have an equivalent bucco‐palatal width; the latter are mesiodistally longer. Porcine molars and premolars have more fissures and grooves that increase their anatomical complexity compared to human molar occlusal surfaces and have more of a mulberry appearance.

### 3.3 Chosen Fluorescence Characteristics

To evaluate fluorescence, the following four emission characteristics were considered.

*Excitation wavelength*. Which excitation wavelength induces maximum fluorescence for both species? The excitation wavelength that induces maximum fluorescence maximizes the signal‐to‐noise ratio and will provide strong emission spectra.

*Fluorescence lifetimes*. How long do fluorescence emissions take to decay? Fluorescence is a luminescence that differs from phosphorescence by an almost immediate cessation of emission upon removal of excitation. Comparing the emission decay following excitation cessation provides another comparable characteristic of fluorescence emissions.

*Fluorescence spectra*. The fluorescence emission spectra arising from each tooth at a given wavelength provide shapes that are visually comparable as a means of assessing similarities and differences of tooth fluorescence.

*Luminosity sensitivity*. To what extent do spectra change shape after initial excitation? Lifetime scans energize samples, which may result in increased or decreased emission intensity by altering a fluorophore's electron cloud. Examining the extent to which spectra differ under cold and energized conditions provides another aspect of emission comparability. This requires a second spectral recording to be performed immediately after the fluorescence lifetime scan.

### 3.4 Process

The teeth were introduced separately into a spectrofluorimeter and orientated such that their thickest enamel was exposed to laser light. For molars and premolars, this was the bucco‐occlusal aspects and for incisors, the labial aspects. These were exposed to 426 nm (see below) pulsed laser light, and the resultant fluorescence spectra were recorded.

The spectrofluorimeter consisted of an F980 Spectrofluorimeter (Edinburgh Instruments company, UK) with an Opotek OPO (Opolette company, USA) for excitation and an air‐cooled R928 (Hamamatsu Company, USA)) photomultiplier for detection. This photomultiplier is silicon‐based, which, like all photomultipliers, has variable sensitivity across its range of detectable wavelengths. As this sensitivity profile is known, recordings were made with its photon count adaptation setting active. This adjusts the actual photon count to compensate for this sensitivity variability to ensure accurate fluorescence photon count recording.

To determine whether the teeth share a common excitatory wavelength that induces maximum fluorescence, 2 nm‐step excitation sweeping scans were initially conducted on a single human and porcine molar, beginning at 410 nm and ending at 500 nm, with the results recorded by the photomultiplier. Once the level of maximum shared excitation was determined, the fluorescence spectra of all twelve teeth were then recorded when illuminated by the OPO at this wavelength.

To determine whether any luminosity sensitivity occurred, each scan was immediately followed by a second scan.

## 4 Results

**Figure 2:**
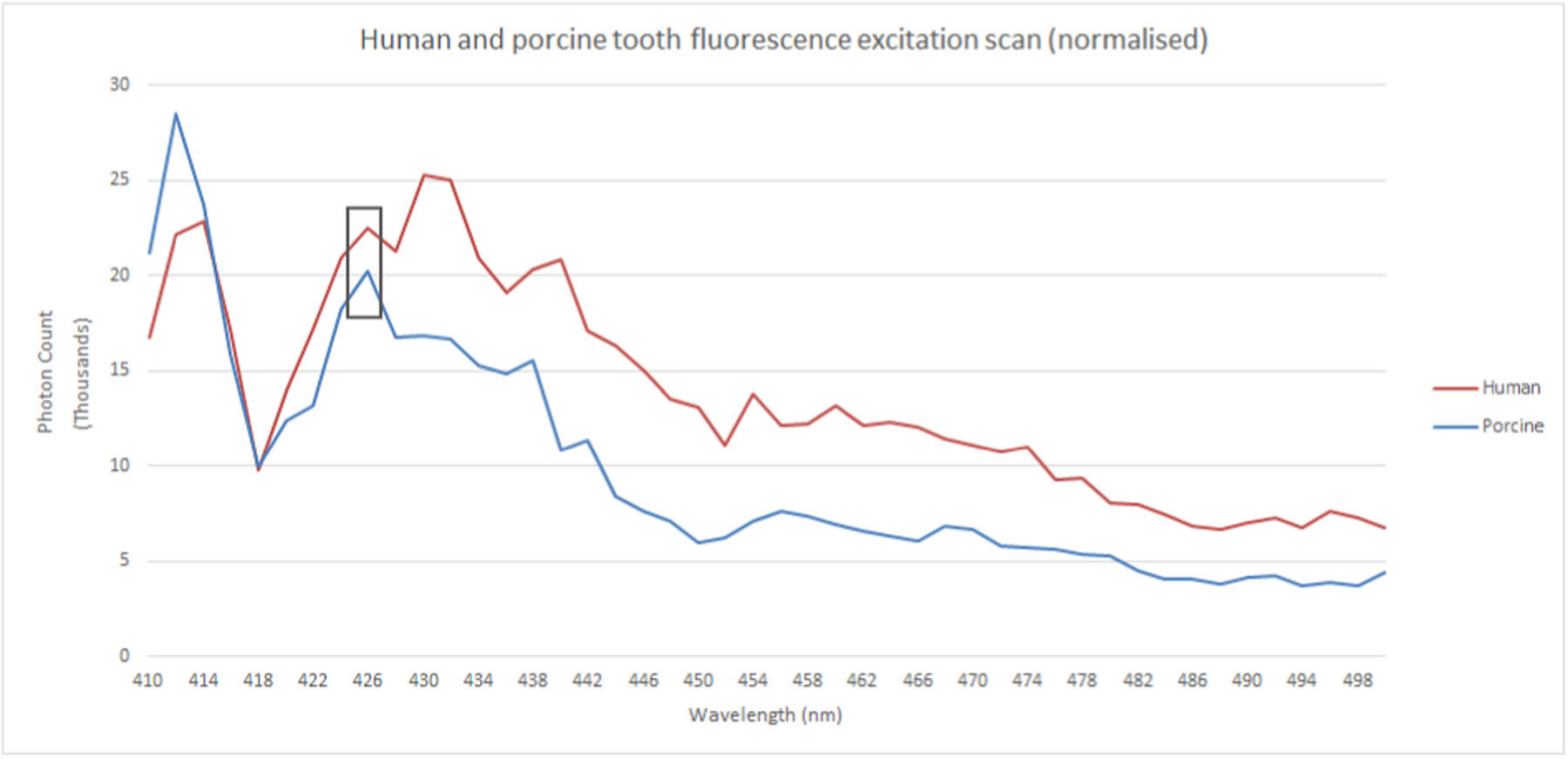
Human and porcine tooth fluorescence excitation scan (normalized).

The results of the initial sweeping scans are shown in Figure 2. While the spike at 411 nm is likely to be an artifact of the spectrofluorimeter, 426 nm (visible violet) gave the greatest response as they coincide as peaks. For excitatory wavelengths longer than this, fluorescence intensity decreases, although emissions continue up to 500 nm, with both sampled teeth demonstrating a comparable decline. This figure suggests that porcine and human teeth share similar profiles of fluorescence emission as the excitatory wavelength lengthens. Note that this does not indicate emission spectra but only overall emission intensity, as it varies with increasing excitatory wavelengths.

As OPO laser energies vary for each output wavelength, the initial excitation scan requires normalizing before peak emissions can be identified. In addition, because this laser energy profile was obtained in 10 nm steps, some interpolation with this 2 nm step excitation scan is also required. This was performed with polynomial regression via R Studio (RStudio Team 2020, RStudio: Integrated Development for R. RStudio, PBC, Boston, MA URL http://www.rstudio.com).

**Figure 3:**
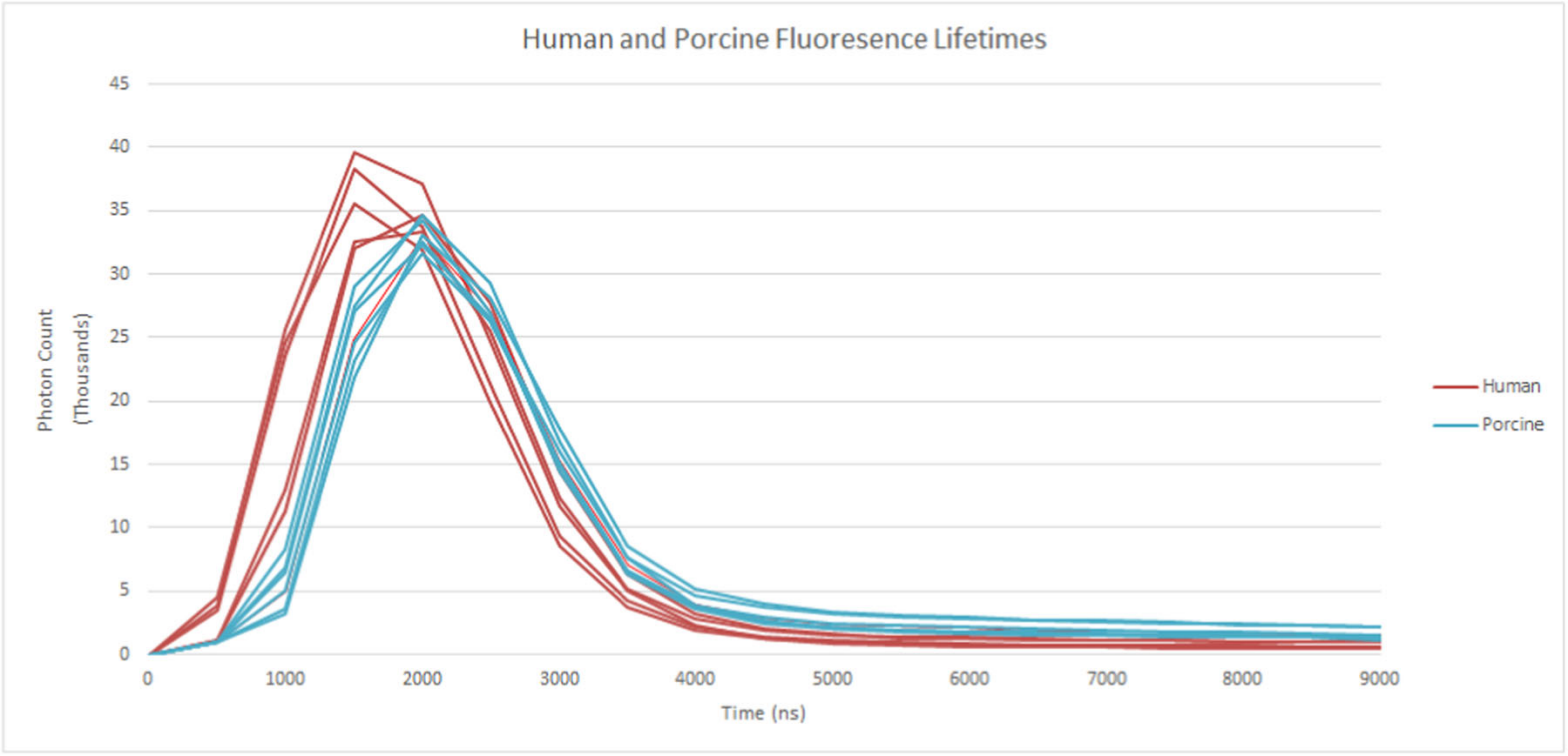
Fluorescence lifetime decay for human and porcine tooth samples at 426 nm excitation.

Fluorescence lifetimes suggest that all teeth appear to share common decay profiles following cessation of excitation at 2000 ns (Figure 3). Although the excitation of human teeth was faster and greater than porcine teeth, porcine teeth showed marginally longer decays. From these decay profiles, it could be argued that both species share similar fluorescence lifetimes. The closer distribution of porcine lifetimes compared to human distributions may be a result of the former being obtained from three animals, while the human teeth have more diverse origins.

While emission decay rates following cessation of excitation were comparable for both species, the human tooth onset rates and intensities were slightly more varied.

**Figure 4:**
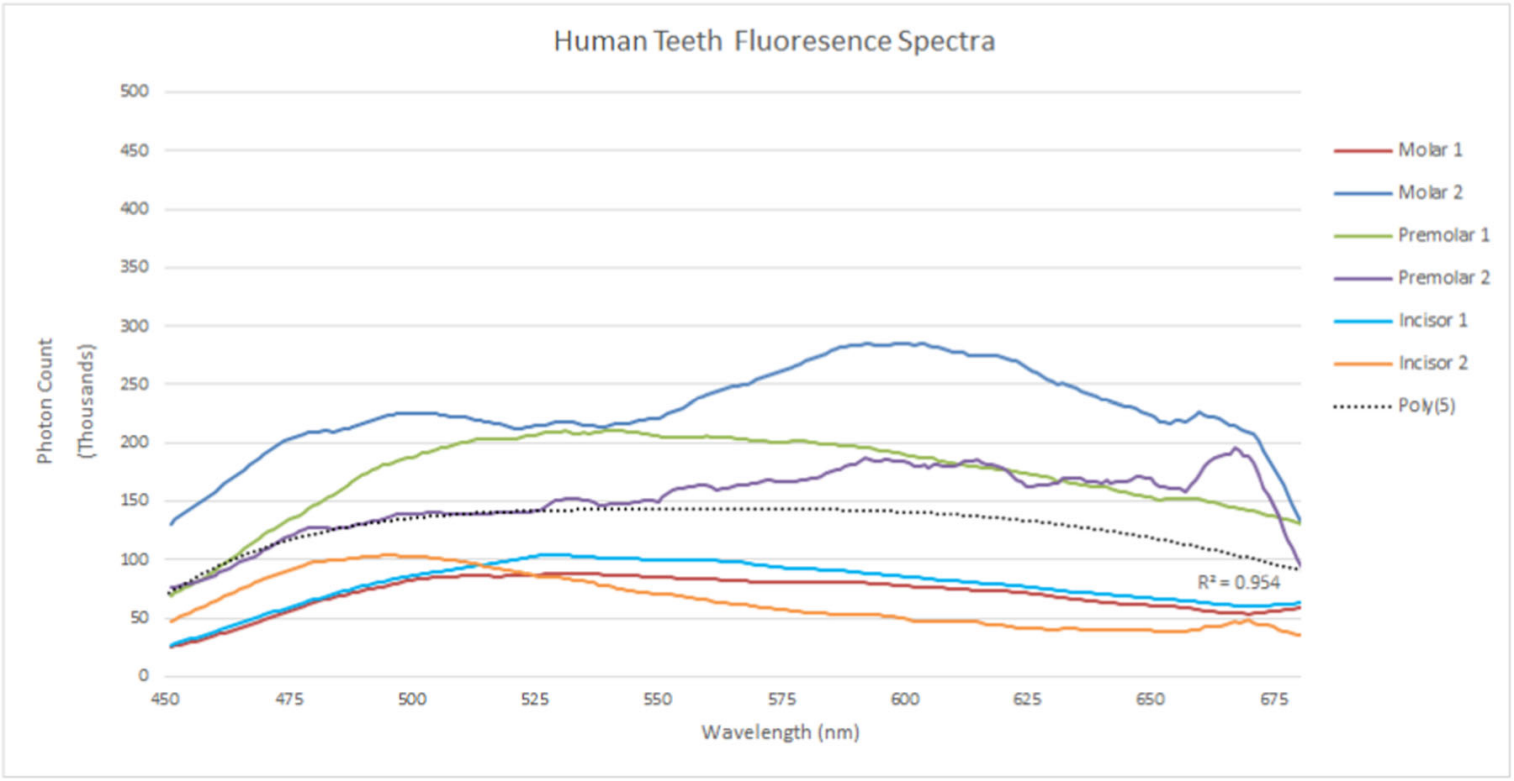
Collective human tooth sample fluorescence spectra.

**Figure 5:**
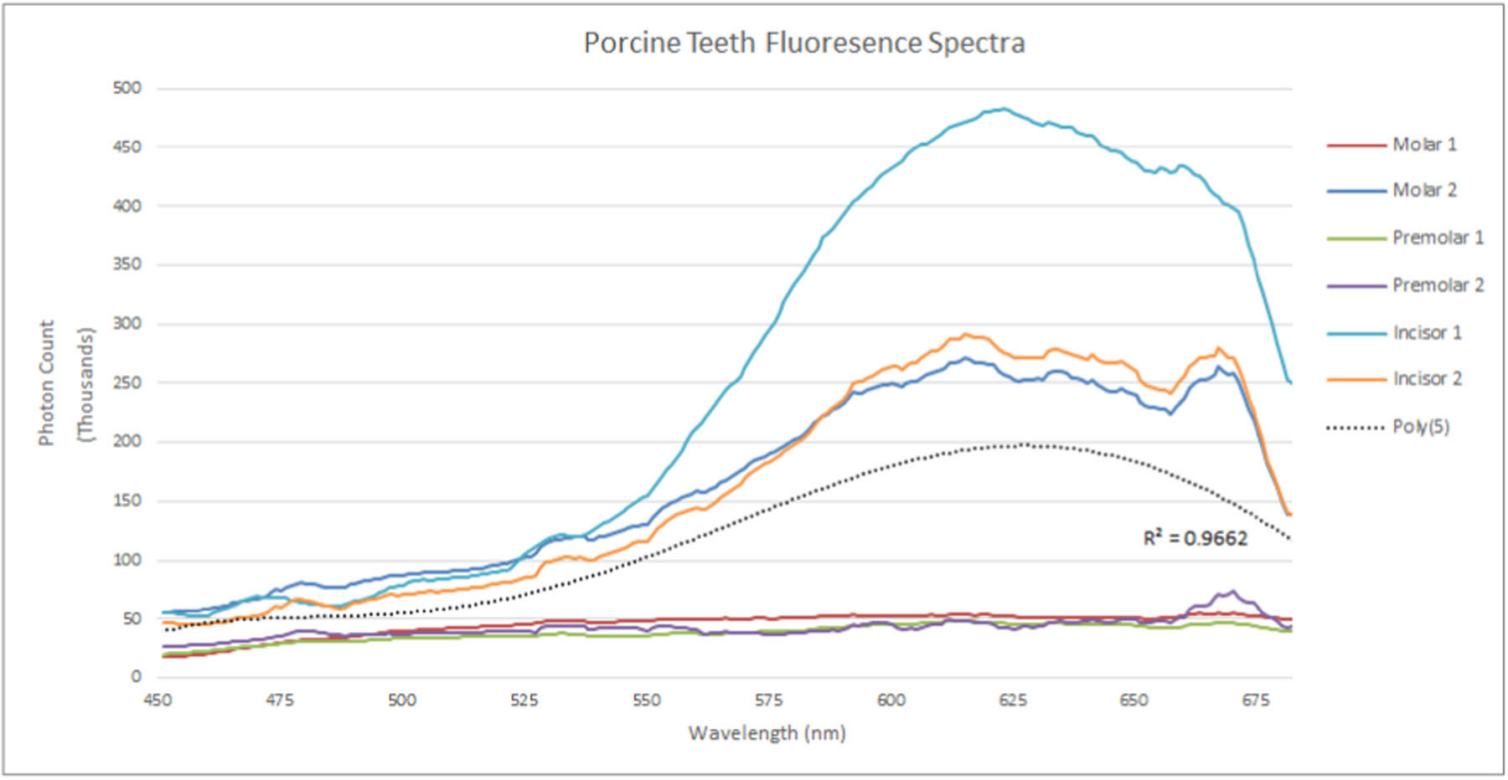
Collective porcine tooth sample fluorescence spectra.

The collective fluorescence spectra from human teeth when excited at 426 nm are demonstrated in Figure 4 and for porcine teeth in Figure 5. While some human and porcine teeth demonstrate similar fluorescence intensities, others show significant disparity. However, there is overall consistency within species with regard to spectral shape. Significantly, human teeth appear to fluoresce across the entire visible spectrum, while porcine teeth showed reduced intensities at shorter emission wavelengths.

Human teeth spectra appear to have bimodal curves, that is, two broad peaks that merge into one across the entire visible spectrum, with one peak spanning 450–530 nm followed by a second from 550 to 675 nm (Figure 4). In contrast, porcine spectra tend to be unimodal, with a broad peak beginning at 550 nm that mimics the second human peak (Figure 5). In other words, while human and porcine teeth have a tendency to share a broad peak from 550 nm onwards, porcine teeth do not appear to have the shorter wavelength spectral portion that human teeth generate.

While human incisors generate relatively less fluorescence than human molars and premolars, the most fluorescent porcine tooth was an incisor. Moreover, while both porcine incisors have similar spectral shapes, one has an intensity double the other. This does not appear to be the case with human teeth. The human molar samples, for example, while having spectra potentially consisting of two broad merging peaks, differ in intensity by a factor of three (Figure 4).

**Figure 6:**
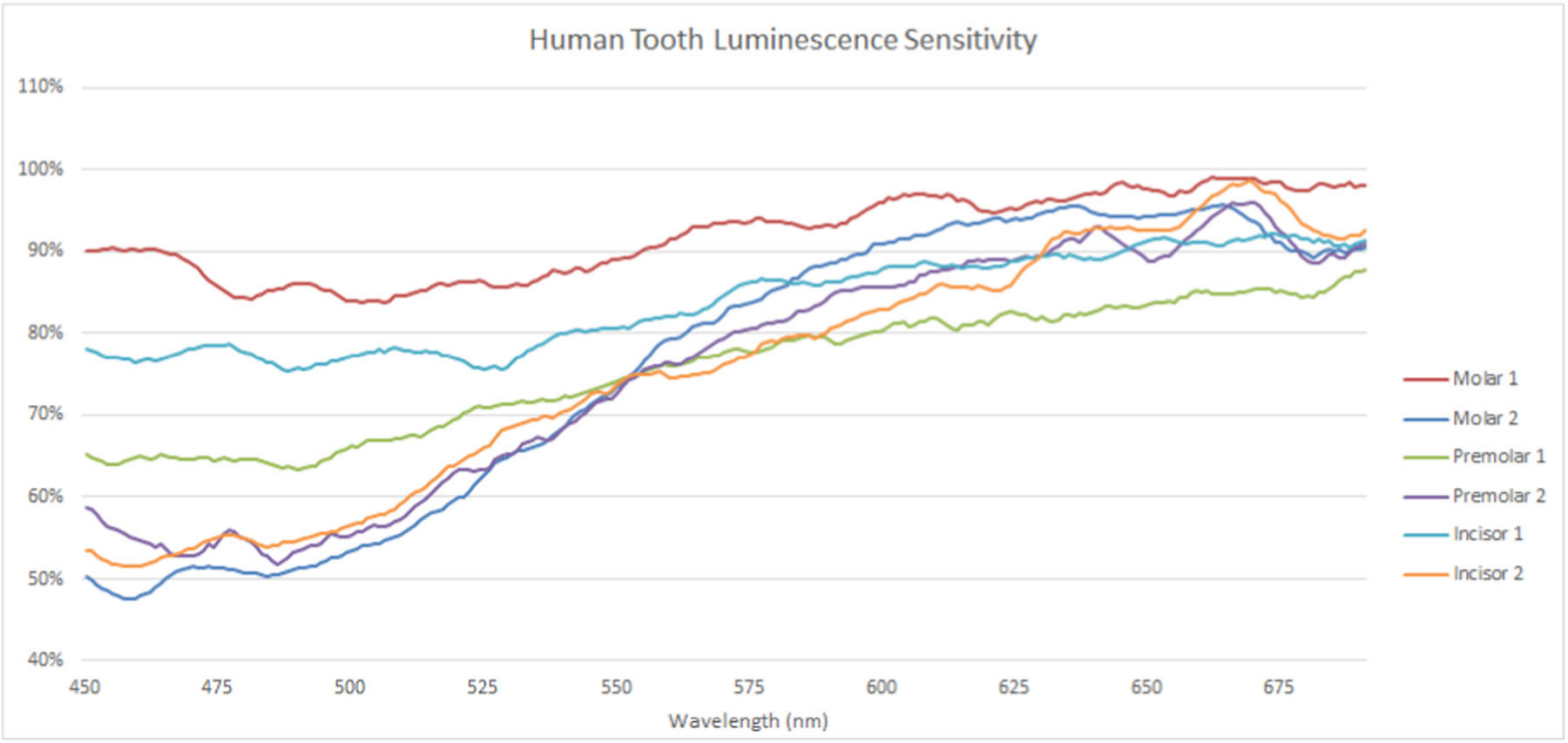
Emissions following the second scan of human teeth. Intensity is demonstrated as a percentage of initial scan results to highlight differences.

**Figure 7:**
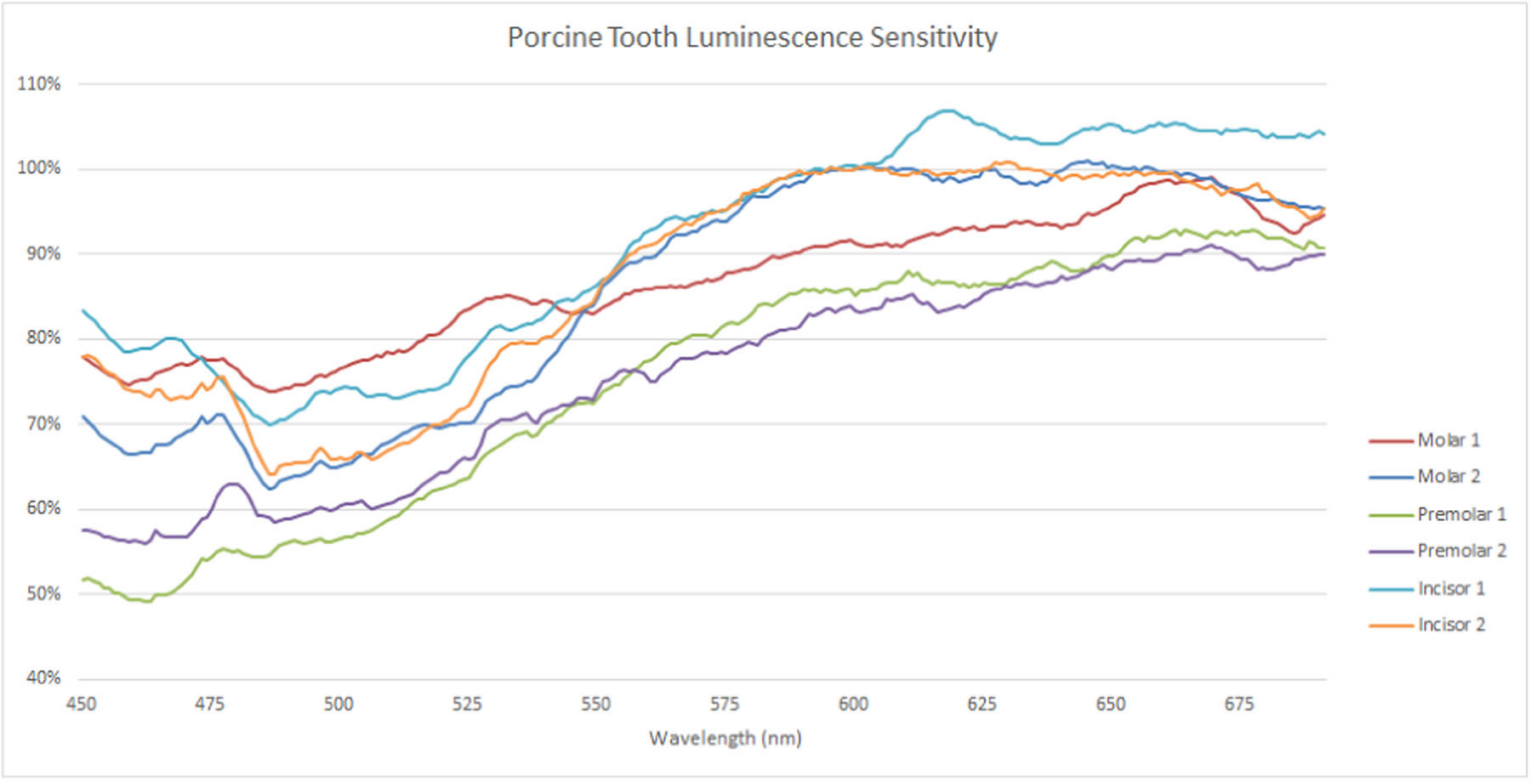
Emissions following the second scan of porcine teeth. Intensity is demonstrated as a percentage of initial scan results to highlight differences.

Luminescence sensitivity, as shown in Figures 6 and 7, appears to affect all teeth of both species to similar extents. Changes in emission intensity occur across the spectra and were seen largely as a reduction in emission intensity. Longer wavelengths were less affected than shorter wavelengths.

## 5 Discussion

This study aimed to compare conventional light‐induced fluorescence between human and porcine teeth under an excitatory visible wavelength that induced the maximum significant fluorescence response in both, as a means of supporting the use of porcine teeth as human analogs for future investigations into dental fluorescence.

While the teeth demonstrated maximum emission intensity at 426 nm excitation, fluorescence of variable intensity occurred across the entire range tested. This concurs with the complexities outlined in the introduction regarding different investigators' definitions of dental fluorescence: that it may range from blue to yellow, depending on the excitatory light (Dickson et al. 1952; Matsumoto, Kitamura, and Araki 1999). A standardized definition of dental fluorescence in terms of color is, therefore, somewhat arbitrary, being dependent on the wavelength inducing it. The fact that each excitatory wavelength induces some fluorescence suggests that a multitude of fluorophores are involved, which may partially explain the difficulty that researchers have in isolating and identifying them. Indeed, even if a particular fluorophore could be isolated for a given excitatory wavelength, for an alternative wavelength, a different fluorophore may be dominant.

The variability in spectral intensity across tooth samples means that fluorescence comparability is better represented by spectral geometry rather than intensity, though reduced intensity does degrade spectral resolution, which impacts its geometry. The technical concerns regarding using a spectrofluorimeter outlined earlier mean that fluorescence intensity is not necessarily a useful characteristic for fluorescence comparison. The quantitative photometry that occurs in such a device is an exacting specialty, and measuring absolute intrinsic fluorescence is technically difficult (Hall, Hefferren, and Olsen 1970; Samokhvalov 2020). The varying geometry of teeth within the fluorimeter's sample chamber results in laser light being incident upon enamel at different angles, which, when coupled with its varying thicknesses, will result in different extents of internal light scattering and reflectivity that affect the amount of fluorescence reaching the detector. This is particularly significant when considering that the excitatory light is both high intensity and pulsed, meaning that all internal layers of teeth will be excited, which may even include residual necrotic pulpal tissue, the organic breakdown products of which may also be fluorescent (Chen et al. 2003). Consequently, characteristics of spectral peak and trough correspondence, rather than intensity, are a better indicator of shared fluorophore characteristics. However, even this comparison needs some caution. The innate translucency of teeth, coupled with dentine's greater proportion of organic content alongside its genetic and structural variability compared to enamel means that its fluorescence is likely to complicate emissions also. This is particularly true of teeth with thinner enamel, such as incisors. Moreover, necrotic pupal remnants are likely to be more prevalent in the recently acquired porcine teeth, despite autoclaving, compared to the decade‐old remnants in the human samples. It might, therefore, be expected that porcine teeth would have broader emission spectra than desiccated human samples, though this does not appear to be the case. Indeed, the genetic, chronological, and environmental consistency of porcine teeth compared to human samples may partially explain the greater spectral consistency of porcine teeth when compared to the more variable human teeth spectra. While human spectra may be influenced by additional intrinsic genetic‐based fluorophores, variability may also arise from additional exogenous fluorophores, such as cumulative minerals and organic aggregates arising from the broader diet, varied environment, oral hygiene practices, and age factors that affect human teeth. For example, these factors may contribute greater zinc, copper, and manganese accumulations in surface enamel, all of which have been shown to have some sensitivity to short wavelength excitation (Matsuura 2017; Reitznerová et al. 2000; Zipkin 1973), while a lifetime of toothpaste use may introduce aluminum, silicas and other zeolites containing alkaline earth metals, which also have fluorescence characteristics (Sahana and Bharadwaj 2014), and all of which will be absent in young, potentially genetically related pigs.

As a consequence, the broader human tooth spectra may arise from numerous contributions of fluorophores, both exogenous and endogenous fluorophores, and may account for differences with the porcine unimodal spectra. It is, however, important to note that these broad spectra differ from other researchers that demonstrated single, shorter fluorescent wavelength peaks, albeit with shorter wavelength excitatory light (Chen et al. 2015). Again, this variability perhaps highlights the sensitivity that tooth fluorescence has to sampling and excitatory conditions. To eliminate internal organic fluorophore contributions, powdered enamel could be examined instead. This would also counter the geometric sample angulation issues mentioned earlier but may not then be directly useful for clinical applications where less intrusive methods are required.

Prolonged exposure at the excitatory wavelength tends to decrease both fluorescence intensity and the spectral shape of both human and porcine teeth. For both species, the majority of this alteration occurs in the shorter wavelength half of spectra. This is apparent despite porcine teeth having less fluorescence intensity in their shorter wavelength spectral portions than human teeth, to begin with, possibly suggesting greater luminescence sensitivity. It could be argued that the decreased fluorescence in this portion of porcine spectra might distort luminescence sensitivity because of lower photon counts, compromising comparability with human teeth that tend to have higher counts in this region. However, the differences appear comparable with human luminescence sensitivities per tooth type, indicating that this characteristic may be related to fluorophore presence, rather than fluorescence intensity. Indeed, both species demonstrated the greatest luminescence sensitivity in their shorter wavelength emissions, which tend to decrease as emission wavelength increases until converging with non‐sensitized emissions. This may be due to prolonged alteration to fluorophore electron valence in response to higher energy emissions that leave the valence in a refractory state. Regardless, both species demonstrate decreased luminosity sensitivity after prolonged exposure to excitatory light, although the degree of desensitization differs, with human teeth demonstrating greater sensitivity than porcine teeth.

Comparing the fluorescence emission lifetimes of human and porcine teeth shows both similarities and differences. A comparable correspondence was seen between species' teeth continuing up to 8 ms after the removal of excitation light. In relation to biological sciences, and in particular to dentistry, luminescence decay within one‐millionth of a second is considered fluorescence, whereas decay longer than this is considered phosphorescence (Walsh and Shakibaie 2007). From this definition, it could be argued that these teeth are demonstrating phosphorescence, as the time from peak intensity to is approximately 1500 nm or 1.5 millionth of a second. Despite this comparability, it could also be argued that, despite their similar shapes, the horizontal difference suggests that human teeth excite faster and with greater intensity than porcine teeth, and decay at similar rates, at least at this resolution.

The primary limitation of this study is the limited number of teeth examined, being 6 from each species and only 2 per tooth type for each species. Despite this, these results nevertheless suggest disparities between both species and tooth types. Despite the normalizing of factors such as consistent size, age, and developmental environment—and the avoidance of human ethics consideration—future investigations into dental fluorescence may be better served by using human teeth rather than porcine samples. This may be particularly relevant to dental material manufacturers attempting to mimic natural tooth fluorescence, which is becoming an increasing consideration in their development.

## 6 Conclusion

Despite the small sample size, these results suggest that, although human and porcine teeth share some luminescence characteristics, they do not have comparable fluorescence spectral geometry at a given excitatory wavelength.

The study highlights notable differences in the fluorescence spectra of porcine and human teeth, though this may be influenced by technique sensitivity. Despite variations in spectral shapes within species, emission intensity is arguably less significant than the spectral shape when comparing these species. Human teeth exhibit bimodal curves with distinct peaks at 450–530 nm and 550–675 nm, while porcine teeth tend to be unimodal with a broad peak starting at 550 nm, lacking the shorter wavelength portion seen in human teeth. Inter‐species fluorescence intensity disparities are observed, with some similarities and differences in spectral shapes among tooth types, suggesting potential genetic influences. Despite variations, luminescence sensitivity affects both species similarly, with reductions in emission across spectra, albeit more consistently in porcine teeth.

Even though human and porcine teeth demonstrate comparable fluorescence lifetime and luminescence sensitivity characteristics, the discrepancies in spectral geometry and intensity for a given excitatory wavelength would suggest that porcine teeth are not acceptable human analogs for dental fluorescence investigations relevant to human teeth.

## Author Contributions

Thomas Corfield and Denice Higgins conceived the study and designed the experiments. Nigel Spooner permitted the experiment in the IPAS labs. Jillian Moffatt and Thomas conducted the experiments and collected the data. Thomas analyzed the data, interpreted the results, and drafted the manuscript. Denice Higgins provided critical feedback and approved the final version of the manuscript.

## Conflicts of Interest

The authors declare no conflicts of interest.

## Data Availability

The datasets generated and analyzed for this study on the difference in fluorescence between human and porcine teeth are available from the author upon request. The datasets generated during the current study are available from the corresponding author on request.
